# α-Synuclein fibrils recruit peripheral immune cells in the rat brain prior to neurodegeneration

**DOI:** 10.1186/s40478-017-0494-9

**Published:** 2017-11-21

**Authors:** Ashley S. Harms, Vedad Delic, Aaron D. Thome, Nicole Bryant, Zhiyong Liu, Sidhanth Chandra, Asta Jurkuvenaite, Andrew B. West

**Affiliations:** 1 Department of Neurology, Center for Neurodegeneration and Experimental Therapeutics, University of Alabama at Birmingham, Birmingham, AL 35294 USA; 2 1719 6th Ave South, Birmingham, AL 35233 USA

**Keywords:** SNCA, Neuroinflammation, Neurodegeneration, Leukocytes, Microglia, Inclusion propagation

## Abstract

Genetic variation in a major histocompatibility complex II (MHCII)-encoding gene (*HLA-DR*) increases risk for Parkinson disease (PD), and the accumulation of MHCII-expressing immune cells in the brain correlates with α-synuclein inclusions. However, the timing of MHCII-cell recruitment with respect to ongoing neurodegeneration, and the types of cells that express MHCII in the PD brain, has been difficult to understand. Recent studies show that the injection of short α-synuclein fibrils into the rat substantia nigra pars compacta (SNpc) induces progressive inclusion formation in SNpc neurons that eventually spread to spiny projection neurons in the striatum. Herein, we find that α-synuclein fibrils rapidly provoke a persistent MHCII response in the brain. In contrast, equivalent amounts of monomeric α-synuclein fail to induce MHCII or persistent microglial activation, consistent with our results in primary microglia. Flow cytometry and immunohistochemical analyses reveal that MHCII-expressing cells are composed of both resident microglia as well as cells from the periphery that include monocytes, macrophages, and lymphocytes. Over time, α-Synuclein fibril exposures in the SNpc causes both axon loss as well as monocyte recruitment in the striatum. While these monocytes in the striatum initially lack MHCII expression, α-synuclein inclusions later form in nearby spiny projection neurons and MHCII expression becomes robust. In summary, in the rat α-synuclein fibril model, peripheral immune cell recruitment occurs prior to neurodegeneration and microglia, monocytes and macrophages all contribute to MHCII expression.

**Electronic supplementary material:**

The online version of this article (10.1186/s40478-017-0494-9) contains supplementary material, which is available to authorized users.

## Introduction

Lewy body diseases like Parkinson disease (PD) are characterized on a pathological level through the presence of protein inclusions enriched in phosphorylated (pSer129) α-synuclein fibrils in susceptible brain regions [12]. In post mortem brain, the abnormal accumulation of immune cells that express major-histocompatibility complex II (MHCII) protein have been described in inclusion-susceptible brain regions [26]. Genome wide association studies have found that genetic polymorphisms in the *HLA-DR* (human MHCII) gene are associated with late-onset PD, implicating innate immune function in disease pathogenesis [15]. MHCII is a crucial regulator of the cellular innate immune response. In response to foreign proteins, the MHCII complex presents antigen to T and B-lymphocytes of the adaptive immune system. This process helps link together innate and adaptive immune responses in disease.

Whereas α-synuclein inclusions in PD localize to neurons, MHCII expression is not neuronal but can occur in microglia and other antigen presenting cells like monocytes from the periphery that can further differentiate into macrophages in the brain. Recent data demonstrate that MHCI expression in neurons may critically mediate α-synuclein neurotoxicity [6]. In contrast with MHCI, the abundance and distribution of MHCII-expressing cells of the innate immune system closely correlates with α-synuclein deposition in neurons [17]. However, the correlation between inclusions and MHCII-expression breaks apart in late stages of disease [7]. In highly susceptible brain regions like the substantia nigra pars compacta (SNpc) and locus coeruleus, the abundance of inclusions diminishes over time due to neurodegeneration whereas the number of MHCII expressing cells is persistent and does not diminish over time [7]. This same phenomenon, persistent MHCII expression long after neurodegeneration, also occurs in the SNpc in individuals exposed to the drug MPTP [8, 22]. Whether MHCII expression is reactive to ongoing neurodegeneration or occurs prior to the loss of cells is hotly contested.

Understanding MHCII responses in the earliest stages of disease, prior to overt cell loss, may divulge the role MHCII expressing cells play in PD. The interpretation of α-synuclein and MHCII-cell recruitment in post-mortem tissue in very early stages of neurodegeneration is controversial because of the lack of clinical diagnosis or prognosis in those subjects. Further, there is a lack of understanding of the cell constituency that accounts for the MHCII induction in PD. Hypotheses that might explain MHCII in PD include 1) a local and profound expansion of resident microglia (e.g., microgliosis) that express MHCII, prior to or after neurodegeneration, 2) recruitment of peripheral MHCII-expressing cells from the periphery at some point in disease, and 3) resident cells in the brain polarize to pro-inflammatory states that upregulate MHCII expression with no expansion and no recruitment of peripheral cells. Resolution of these possibilities may be critical for successfully targeting these changes in the brain for therapeutic benefit.

Model systems in rodents may be useful for addressing these open questions and are ideal for studying very early changes in the neurodegenerative process where outcomes are clearer. MHCII cell activation has been described in several models of PD including 6-OHDA lesions and viral-mediated over-expression of α-synuclein [19, 20, 34]. In newer approaches to model PD, it has been demonstrated that short α-synuclein fibrils prepared in vitro can be applied to neurons leading to their uptake, intracellular spread, and eventual seeding activity that results in intraneuronal inclusions [24, 41, 42]. Whether fibril exposures and inclusion formation are accompanied by an MHCII response like that found in PD has not been previously described. Recently we developed a variation of the α-synuclein fibril model in rats where inoculation of very-short fibrils directly into the SNpc causes inclusion formation in tyrosine-hydroxylase (TH)-expressing neurons [1]. Here, we utilize the excellent immunological tools and antibodies developed for rat models of inflammatory disease to probe MHCII-expression and related changes in neuroinflammation profiles at different timepoints. We find that α-synuclein fibrils, but not monomer, sets off a cascade of MHCII-expression in the SNpc composed of both microglia and peripheral monocytes and macrophage responses. MHCII expression, like in PD, does not disappear with time, but spreads outward from the SNpc. Assuming the rat model used here is relevant to PD, these results provide evidence that the MHCII response associated with α-synuclein involves both microglia (that do not expand) and peripheral monocytes (that are recruited) prior to neurodegeneration.

## Materials and Methods

### Generation of α-synuclein fibrils, biosafety, and biophysical measures

Mouse α-synuclein, encoded in pRK172 was purified from BL21 (DE3) Codon Plus cells (Clontech). Bacterial growth was monitored to log-phase, IPTG added for 2 hours at 37°C, paste collected into lysis buffer consisting of 750 mM NaCl, 10 mM Tris, pH 7.6, 1 mM EDTA, 1 mM PMSF, and 1x bacterial protease inhibitor cocktail (RPI). Homogenates were sonicated and tubes placed in boiling water for 15 minutes. After centrifugation for 25 minutes at ~10,000 x g, samples were loaded into tubing (3.5 kDa MWCO, Fisher) and dialyzed into 10 mM Tris, pH 7.6 with 50 mM NaCl, 1 mM EDTA, PMSF. Supernatant were next centrifuged at 100,000 x g for 1 hour at 4 C, and concentrated using Amicon Ultra15 3.5 MWCO columns. Concentrates were separated through a HiLoad 16/600 Superdex Column, 1 x 120ml (GE Healthcare), samples with α-synuclein dialyzed and concentrated again, and separated through a HiPrep Q HP 16/10 Column, 1 x 20 mL (GE Healthcare). α-Synuclein was eluted from the column with a gradient application of high-salt buffer, and samples containing α-synuclein were dialyzed, applied to a high-capacity Endotoxin Removal spin column (Pierce), and elute concentrated a third time. Concentration of monomeric protein was determined by BCA assay (Pierce).

α-Synuclein fibrils were generated by 700 R.P.M. shaking a microcentrifuge tube containing 5 mg mL^-1^ monomer α-synuclein for seven days at 37^o^C. Fibrils were sheared with sonication (Fisher Scientific Sonic Dismembrator FB120110). Since sonication aerosolizes fibrils, all sonication steps were performed in a biosafety-level 2 cabinet with the operator wearing disposable wrist guards and double-gloved. Clean-up and inactivation of the fibrils on contaminated surfaces was accomplished using a 1% SDS solution as described [3, 39].

For circular dichroism, 0.1 mg mL^-1^ of monomer or fibril samples were loaded onto a photospectrometer cell with a 0.2 mm path length and scanned with the CD DSM-20 system (Olis), with a wavelength from 190 to 260 nm. For dynamic light scattering, estimated molecular weight of monomers and sonicated fibrils, 0.05 mg mL^-1^ of α-syn samples were measured in microcuvettes with a DynaPro NanoStar (Wyatt Technology) at 25°C, with data collected and analyzed using Dynamics software. For transmission electron microscopy, 3 μL of 0.1 mg mL^-1^ samples were applied to glow-discharged 400 mesh, carbon-only, copper grids (Electron Microscopy Sciences) and negatively stained with 1% uranyl acetate (Polysciences). The grids were imaged in a FEI Tecnai F20 electron microscope (Eindhoven) operated at 200 kV with nominal magnification at 65,000x and a defocus range of -1.0 μm to -1.27 μm. Images were collected on a Gatan Ultrascan 4000 CCD camera. Endotoxin levels were determined using an LAL chromogenic endotoxin quantification kit (Pierce). Immediately prior to usage of fibrils or monomer protein in experiments, monomeric protein was subjected to ultracentrifugation 100,000 x g for one hour, and supernatants containing either monomer the prepared fibril α-synuclein protein was determined using a NanoDrop instrument with 7.45 as the extinction coefficient and 14.45 as molecular weight.

### Primary microglia culture

All animal protocols were approved by the University of Alabama at Birmingham Institutional Animal Care and Use Committee. Timed-pregnant C57BL/6J mice were obtained from Jackson Laboratories and P0-3 pups were isolated, meninges removed, and brain tissue dissociated and filtered (0.2 micron). Filtrate was plated into media containing 20% heat inactivated fetal bovine serum, penicillin/streptomycin, and L-Glutamine (Sigma), and 10ng mL^-1^ granulocyte monocyte colony stimulating factor (PeproTech). Cells were maintained for two weeks and microglia isolated by mechanical shaking at 195 R.P.M. for 1 hour at 37C and plating to new wells.

For DQ Ovalbumin measurements, microglia were treated with fibrils or monomer for 30 minutes followed by the addition of DQ Green BSA (Molecular Probes) for one hour. Anti-iNOS (Abcam) and anti-MHCII (clone M5/114.15.2, eBiosciences) were used to stain microglia as previously described (Harms 2013). Images were captured with a Lecia TCS-SP5 confocal microscope and intensities were quantified using ImageJ software. Conditioned media was collected from each experiment and supernatants analyze with the Milliplex Mouse Cytokine/Chemokine Magnetic Bead 25 Plex Kit (MD Millipore)

### Injections, immunofluorescence and immunohistochemistry

Intracranial injections of fibrils or monomer control (equivalent w/v) were performed in 100 Sprague Dawley rats (Taconic Farms) aged ~8-10 weeks on a digital stereotaxic frame (David Kopf). Proteins were injected into the rats at the SNpc at 4.65 mm posterior and 2.25 rat mm lateral to Bregma, and 7.45 rat mm ventral relative to the skull). In all experiments, 4 μL of saline, or saline that included 8 μg of monomeric α-synuclein, or 8 μg of matched (same batch of protein as monomer) short-α-synuclein fibrils (concentrations determined by A_280_ prior to injection via Nanodrop analysis) were injected. Rats were transcardially perfused with PBS (pH 7.4). For immunohistochemistry and imaging experiments, this was followed by freshly prepared 4% PFA buffered in PBS. Brains were removed, post-fixed for 24 hours in the 4% PFA and PBS solution, floated into 30% sucrose PBS solution for up to three days, and frozen in isopentane solution (-50 C).

Sections were cut to 40 μm on a freezing microtome and incubated in an antigen recovery solution of 10 mM sodium citrate, pH 6.0, supplemented with 0.05% tween, at 37 °C for 1 hour with gentle rocking. Sections were rinsed with tris-buffered saline (TBS, pH 7.4) and combined with blocking buffer (5% normal goat serum with 0.3% Triton X-100 in TBS). Sections were rinsed and combined with primary antibodies that include pS129-α-synuclein (clone 81A, Biolegend), NeuN (Clone A60, IgG1, EMD Millipore), tyrosine hydroxylase (rabbit polyclonal, EMD Millipore), IBA-1 (rabbit polyclonal, Wako Chemicals), CD613 (Clone ED2, Biorad), and MHCII (Clone RT1B, BD Bioscience). Sections were rinsed and combined with secondary antibodies for 24 hours at 4 °C with gentle rocking. Sections were mounted to superfrost slides and mounted to coverslips with Prolong Gold (Invitrogen). Confocal microscopy was carried out with a Leica SP5 and imaging processing with LAS X software (Leica).

For immunohistochemistry with 3,3′-diaminobenzidine (DAB) and Nissl staining, brain sections were treated as above except for a quench for 30 min at room temperature with 0.3% H_2_0_2_ in methanol following antigen retrieval. Secondary antibodies (Jackson Immunologic), conjugated to biotin were used and sections treated with the ABC kit (Vector) followed by development with Impact DAB reagent (Vector). Sections were dehydrated progressively in ethanol and then Histo-Clear (national Diagnostics) and mounted to coverslips with Permount reagent (Fisher). Some sections were rehydrated with immersion to water and then treated with cresyl violet solution (0.1% cresyl violet, 0.08% acetic acid) for one-minute in a microwave, and then rinsed in water and dehydrated with ethanol, and immersed back into Histo-clear and mounted onto slides. Slides were analyzed on an Olympus BX61 wide field microscope.

### Mononuclear cell isolation and flow cytometry

Intracranial injections of fibrils or monomer control (equivalent w/v), or saline, were performed in 45 Sprague Dawley rats (Taconic Farms) aged ~8-10 weeks on a digital stereotaxic frame (David Kopf). Brain tissue punches from the SNpc or striatum were dissected 8 weeks after injections. Individual samples were formed by pooling (three rats each) SNpc punches, or dorsal striatum, each from different animals. In each experiment, treatment groups (PBS, monomeric alpha-synuclein, fibril alpha-synuclein) consisted of five of these independent sample pools. Mononuclear cells were isolated using enzymatic digestion with 1 mg mL^-1^ Collagenase IV (Sigma) and 20 μg mL^-1^ DNAseI (Sigma) diluted in RPMI 1640 with 10% heat inactivated fetal bovine serum and 1% L-glutamine from the pooled ventral midbrains or striatum of rats injected with PBS, monomeric α-synuclein, or α-synuclein fibrils. Mononuclear cells were isolated eight weeks post transduction via 30/70% percoll gradient as previously described [33]. Isolated cells were first blocked with an Fcγ receptor solution (1:500 w/v), surface stained for CD45 (OX-1), CD11b/c (OX-42), RT1D (OX-17), CD4 (W3/25), and CD8 (OX-8), all from Biolegend as previously described [33]. Stained cells were analyzed using an Attune NxT (Thermo Fischer Scientific) and data analyzed using FlowJo software. Gating scheme is provided in Additional file 1: Figure S2. In each experiment, live cells were first gated using a fixable viability dye (Vibrant Aqua eBioscience 1:1000) per the manufacturer’s instruction, followed by CD45. This gating scheme allows for selection of all immune cells first including T cells, followed by CD11b to subdivide myeloid subsets.

### Unbiased approaches, stereology, and statistics

Unbiased stereological estimations of the total number of TH+ (Nissl counterstain) in the SNpc was performed using an optical fractionator probe (*Stereologer* software, Stereology Resource Center) by an investigator blinded to the experiment identity. Sections used for counting covered the entire SNpc and were equally spaced 120 μm apart, with random frame placement through all counting areas. Guard zone height was 2 μm to avoid artifacts on the cut surface of sections. Sampling grid size (e.g., 200 μm) was adjusted as needed, due to the heterogeneity of lesion size, to allow up to five objects on average counted at each of 100 to 200 x-y locations (minimum 100 objects counted for each observation, based on mean section thickness). Cells that were large (~30-50 μm) and Nissl+ but not TH positive within the borders of the SNpc were also counted (less than 1% of the total counts in each experimental group reported herein). For counting soma inclusions through the SNpc or striatum, a rare-object counting probe was utilized. Only somatic perinuclear (e.g., adjacent to Nissl bodies) phosphorylated α-synuclein inclusions were counted.

Statistical analysis and graphs were performed and created with Graphpad Prism 5.0 software. Two-tailed one-way ANOVA tests were used to calculate p values, where less than 0.05 was considered significant, and for those groups with significant differences, a post-hoc test (Tukey’s) was applied to each possible combination. Linear regression lines are shown for some plots.

## Results

### α-Synuclein fibril exposure promotes antigen processing, presentation and activation in primary microglia

Aggregated forms of α-synuclein may activate primary cultured microglia through toll-like receptor signaling pathways [11, 37]. Very short α-synuclein fibrils have been identified as conformers that can seed the formation of new inclusions in neurons, and eventually cause progressive dopaminergic neurodegeneration in rats [1, 29]. We wondered whether the concentrations and compositions of short-fibrils we and others have used in primary neurons to induce inclusions might cause microglia activation phenotypes similar to responses observed with applying large α-synuclein aggregates and fibrils to microglia [14, 20, 21].

**Fig. 1:**
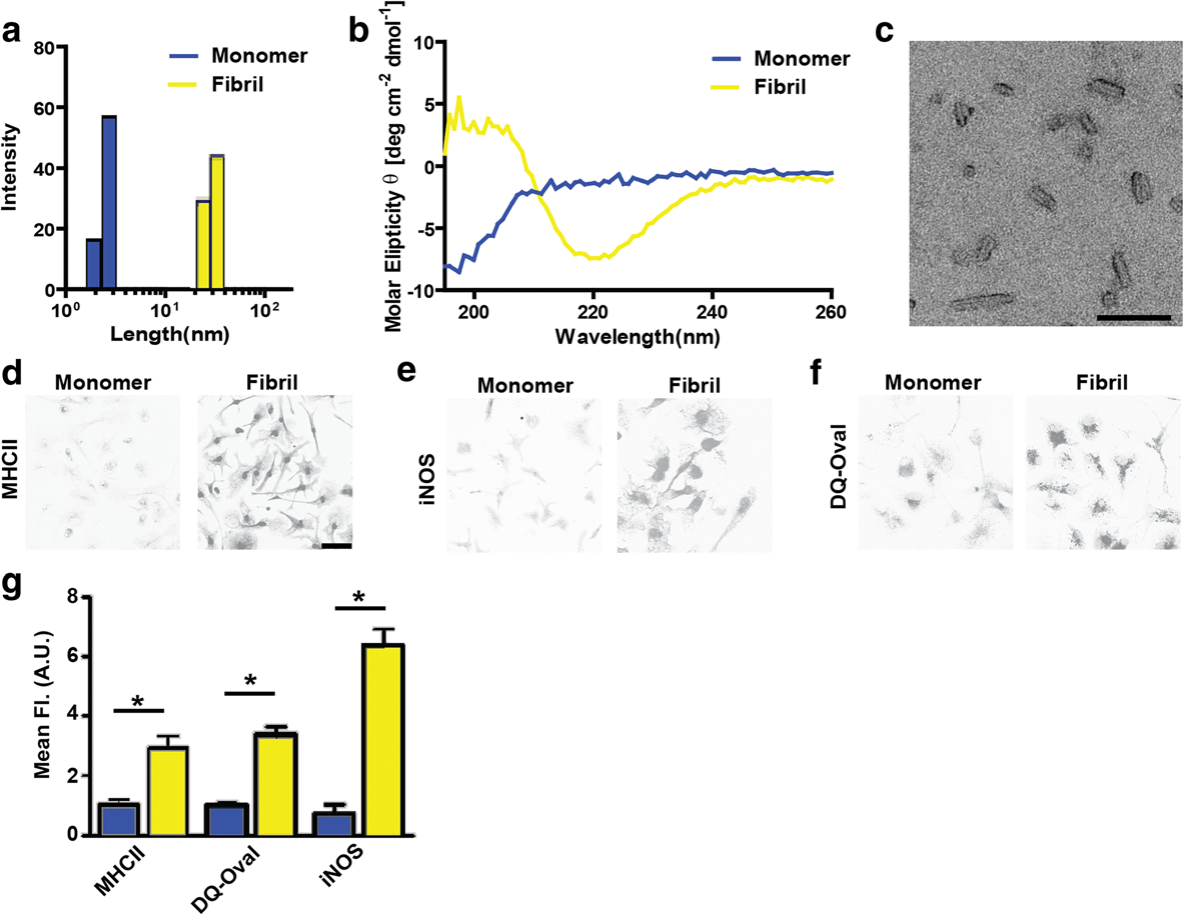
α-Synuclein fibril exposure promotes antigen processing, presentation and activation in primary microglia. **a** Dynamic light scattering analysis and **b** circular dichroism of monomeric (blue) and fibril (yellow) preparations of α-synuclein. **c** Representative electron microscopy photomicrograph of α-synuclein fibrils. Scale bar is 100 nm. **d** Representative confocal images of MHCII and **e** iNOS immunohistochemistry of primary microglia cells treated with 1 μg mL^-1^ α-synuclein monomer or fibrils for four hours. **f** DQ-Ovalbumin fluorescent images of primary microglia treated with α-synuclein monomer and fibrils for thirty minutes prior to imaging. Scale bar is 50 μm. **g** Quantification of MHCII, iNOS, and DQ-Ovalbumin fluorescence in primary microglia. Mean values are calculated from at least four different photomicrographs from three independent experiments. **p<0.05, one-way ANOVA and Tukey’s post hoc test

To generate short fibrils and matched monomer preparations (protein from the same catch of starting recombinant protein), we purified recombinant α-synuclein from bacteria and depleted endotoxin content with reduced poly-lysine columns. Next, we verified preparations of either monomeric (depleted of aggregates via ultracentrifugation) or sonicated short fibrils using dynamic light scattering (Fig. 1a). The fibril preparation had an average length of ~30 nm compared to monomeric protein (average length of ~2 nm). Larger α-synuclein conformers greater than 2 nm in the monomeric preparation could not be detected by light scattering. Based on A_280_ measurements of protein concentration, the amount of α-synuclein protein did not vary in the monomeric or short-fibril preparations. Endotoxin as measured through Limulus Amebocyte Lysate testing revealed a concentration of 14.9 endotoxin units (EU) per milligram (i.e., 0.015 EU μg^-1^) of protein common to the preparations of monomer, unsonicated fibrils, and short-fibrils. Circular dichroism easily distinguished the monomer protein from the fibrils based on β-sheet conformations inherent to α-synuclein protein fibrils (Fig. 1b). Electron microscopy of the fibril preparation corroborated the light scattering-predicted length of the fibrils (Fig. 1c).

To determine whether monomeric or short α-synuclein fibrils might provoke differential effects in microglia, we treated mouse primary microglia with monomer or fibrils using matched amounts of α-synuclein protein (based on A_280_) with amounts of fibrils that can result in inclusions in primary neurons (~1 nM fibrils, see [1]). After four hours, we measured MHCII and iNOS expression that are both canonical markers of pro-inflammatory activation and found that the fibrils induced these markers three to six-fold (respectively) over monomer α-synuclein exposures (Fig. 1 d, e). To determine whether the fibrils could increase antigen processing and presentation, DQ ovalbumin was added to the primary microglia cultures for one hour. Following the addition of α-synuclein fibrils to primary microglia for thirty minutes, a marked induction of processing was observed via fluorescence emitted from cleaved DQ ovalbumin (Fig. 1f,g), consistent with MHCII upregulation.

To further establish that α-synuclein fibrils provoke microglia activation as predicted by iNOS and MHCII expression, we evaluated secreted cytokines and chemokines two and four-hours after fibril and monomer exposures. Several increased soluble factors associated with pro-inflammatory responses were detected (Table 1). The most impressive change that we could measure was the induction of IL-6, a pro-inflammatory cytokine that may be elevated in PD cerebral spinal fluid and plasma [9, 10], that changed from ~3 pg mL^-1^ in concentration in monomer α-synuclein exposures to ~1 ng mL^-1^ four-hours after α-synuclein fibril exposure (over three-hundred-fold induction). Other subtler increases caused by fibril exposures included granulocyte-colony stimulating factor (G-CSF), interleukin-1 α (IL-1α), interleukin-17 (IL-17), and tumor necrosis factor (TNF). In control wells not exposed to α-synuclein protein, these cytokines were below our limits of detection (<1 pg mL^-1^). Microglia also expressed heightened levels of chemokines caused by the fibril exposures and these included IP-10, KC, MCP-1, MIP-1α, MIP-1β, and MIP-2 (Table 1). Overall these data suggest a pro-inflammatory effector function by microglia elicited by short fibril conformations of α-synuclein. Equivalent amounts of monomer α-synuclein that contained equivalent concentrations of endotoxins and other co-precipitating factors failed to induce these responses, demonstrating the requirement of the fibril-conformations of α-synuclein to evoke MHCII-expression.

**Table 1:** Primary microglia treated with ~1 nM concentration of α-synuclein fibrils (see Fig. 1), or the equivalent amount (w/v) of monomeric α-synuclein, for the indicated amount of time

Analyte (pg mL^-1^)	Monomer (2 hours)	Fibril (2 hours)	Fold (fibril/monomer)	p*	Monomer (4 hours)	Fibril (4 hours)	Fold (fibril/monomer)	p*
G-CSF	0.70±0.38	3.71±0.26	5.33±0.37	0.0028	0.94±0.62	45.32±8.02	65.06±11.51	0.0053
IL-1α	18.49±1.74	30.38±2.76	1.64±0.15	0.022	16.78±1.76	85.06±10.16	5.07±0.61	0.0027
IL-2	1.06±0.14	1.45±0.26	--	ns	0.82±0.08	1.54±0.09	1.88±0.11	0.004
IL-4	0.29±0.02	0.27±0.02	--	ns	0.29±0.02	0.34±0.02	--	ns
IL-5	1.23±0.40	1.88±0.26	--	ns	1.33±0.33	2.92±0.64	--	ns
IL-6	2.78±0.86	84.07±6.48	30.20±2.33	0.0002	2.87±1.84	1000±286.4	348.57±99.77	0.0253
IL-9	13.92±5.96	3.62±3.62	--	ns	11.99±1.33	10.43±1.41	--	ns
IL-10	1.14±0.28	0.96±0.51	--	ns	0.46±0.14	3.79±1.01	8.29±2.20	0.0306
IL-15	0.83±0.83	2.08±1.76	--	ns	2.28±1.26	9.69±3.71	--	ns
IL-17	0.37±0.04	0.61±0.07	1.63±0.20	0.0456	0.32±0.03	1.77±0.20	5.59±0.64	0.0021
IP-10 (CXCL10)	2.55±0.23	24.23±1.34	9.51±0.52	<0.0001	4.20±1.42	242.5±56.68	57.68±13.48	0.0137
KC (CXCL1)	8.00±1.21	93.83±4.55	11.72±0.57	<0.0001	8.68±2.86	635.1±119.7	73.20±13.80	0.0064
MCP-1 (CCL2)	428.1±55.54	470.4±76.83	1.10±0.18	0.0126	698.3±29.25	1415±175.7	2.03±0.25	0.0079
MIP-1α	163.6±14.40	365.2±2.08	2.23±0.01	0.0002	194.9±23.25	1739±343.2	8.93±1.76	0.0109
MIP-1β	181.3±21.37	590.7±19.38	3.26±0.11	0.0001	237±41.94	4729±734.6	19.95±3.10	0.036
MIP-2	50.94±3.13	750.3±55.95	14.73±1.10	0.0002	85.52±20.56	3237±232.7	37.85±2.72	0.0002
RANTES	1.08±0.11	1.22±0.09	--	ns	0.98±0.09	8.12±2.40	8.29±2.45	0.0412
TNF	6.52±0.88	248.4±10.39	38.08±1.59	<0.0001	8.85±1.69	754.7±103.3	85.28±11.67	0.002

*ns is non-significant

*P* values were calculated with one-way ANOVA tests with Tukey’s post-hoc analysis

### α-Synuclein fibril exposure in the rat SNpc induces a rapid MHCII response

**Fig. 2:**
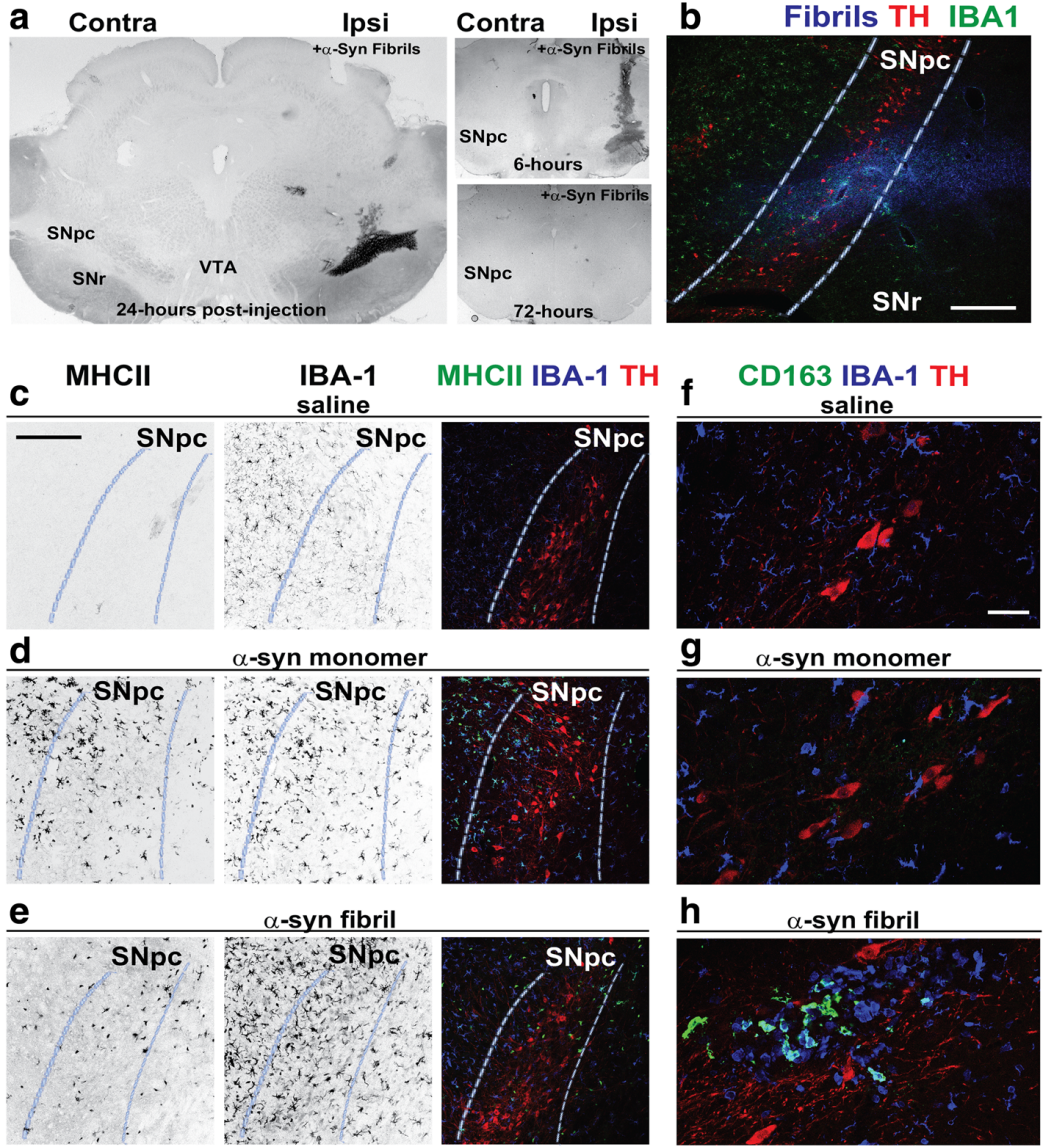
α-Synuclein fibril exposure in the midbrain induces a rapid innate immune response. **a** Immunohistochemisty for SYN1 (α-synuclein, NiDAB-black) to label injected α-synuclein fibrils 6, 24, or 72 hours post-fibril injection, as indicated. Brain regions are depicted as follows: SNpc, substantia nigra pars compacta, VTA, ventral tegmental area, SNr, substantia nigra pars reticulata. **b** Fluorescent immunohistochemistry depicting α-synuclein fibril spread (SYN-1, blue color) and microglial localization (IBA-1, green color) in the ipsilateral SNpc (highlighted with dashed white/blue lines) with TH as red color, 24 hours post injection. Confocal image, Scale bar is 0.2 mm. **c** Immunohistochemistry depicting inflammation in the ipsilateral SNpc of saline, **d** monomer, or **e** fibril-injected rats, 24-hours post injection. Representative individual channels (depicted in grayscale for clarity) and merged images are shown (merged is MHCII-green, IBA-1-blue, TH-red). Scale bar is 150 μm. **d** Immunohistochemistry depicting inflammation in the ipsilateral SNpc of monomer injected rats, 24 hours post injection. Individual channels and merged confocal images are shown (MHCII-green, IBA-1-blue, TH-red). **e** Immunohistochemistry depicting inflammation in the ipsilateral SNpc of α-synuclein fibril injected rats, 24 hours post injection. Individual channels and merged confocal images are shown (MHCII-green, IBA-1-blue, TH-red). **f** Immunohistochemistry depicting peripheral CD163+ myeloid infiltration and inflammation in the ipsilateral SNpc of saline, **g** monomer, or **h** fibril-injected rats, 24 hours post injection. Representative confocal images are shown for CD163 staining that is outside the vasculature (i.e., perivascular CD163+ macrophages are not shown). CD163 is green color, IBA-1-blue color, TH-red color. Scale bar is 150 μm. Images are representative of at least three rats analyzed per group

To determine whether α-synuclein fibril exposures could provoke rapid inflammation phenotypes in the brain as suggested by our *in vitro* studies with primary microglia (Fig. 1), we injected 4 μL of saline, or saline that included 8 μg of monomeric α-synuclein, or 8 μg of matched (same batch of protein as monomer, both with ~0.12 total EU included) α-synuclein fibrils into the rat SNpc. In serial sections across the midbrain, the injected fibrils could be detected by immunohistochemistry and rapidly spread across the entirety of the SNpc as observed twenty-four hours after injection (Fig. 2a). By 72 hours post-injection, the fibril material could not be detected in the SNpc. To determine microglial activation profiles in the SNpc in the presence of the injected fibrils or monomer protein at 24 hours, as well as inflammation caused by the surgery and saline injections alone, we immunostained sections of midbrain with MHCII to probe pro-inflammatory responses, IBA-1 to identify resident microglia, microgliosis (expansion of microglia, and/or reduction of turnover), and tyrosine hydroxylase (TH) for dopaminergic neuron identification. IBA-1 expression appeared upregulated 24-hours after injections in all conditions when compared to the contralateral side, consistent with effects due to the surgery alone (Fig. 2c-e). The saline injections failed to induce any MHCII expression. In contrast, both monomer and fibril α-synuclein upregulated MHCII in many of the ramified IBA-1 cells (Fig. 2c-e). We noticed that fibril injected rat sections often harbored a proportion of MHCII-positive cells that were much weaker in IBA-1 expression and had amoeboid morphology distinct from typical microglia (Fig. 2e). CD163 is a scavenger receptor demonstrated to demarcate monocytes and macrophages from the periphery [31], and these cells poorly express IBA-1 but can robustly express MHCII. We could not detect CD163-positive cells in the SNpc of saline or monomer α-synuclein injected rats, but all the fibril injected rats demonstrated robust CD163 staining (Fig. 2h). These results demonstrate that all conditions including surgery alone active the innate immune response to some extent, but α-synuclein fibrils were unique in recruiting MHCII-positive IBA-1 weak and CD163-positive cells within the fibril injection field.

### α-Synuclein fibrils but not monomer result in a sustained MHCII response in the SNpc

**Fig. 3:**
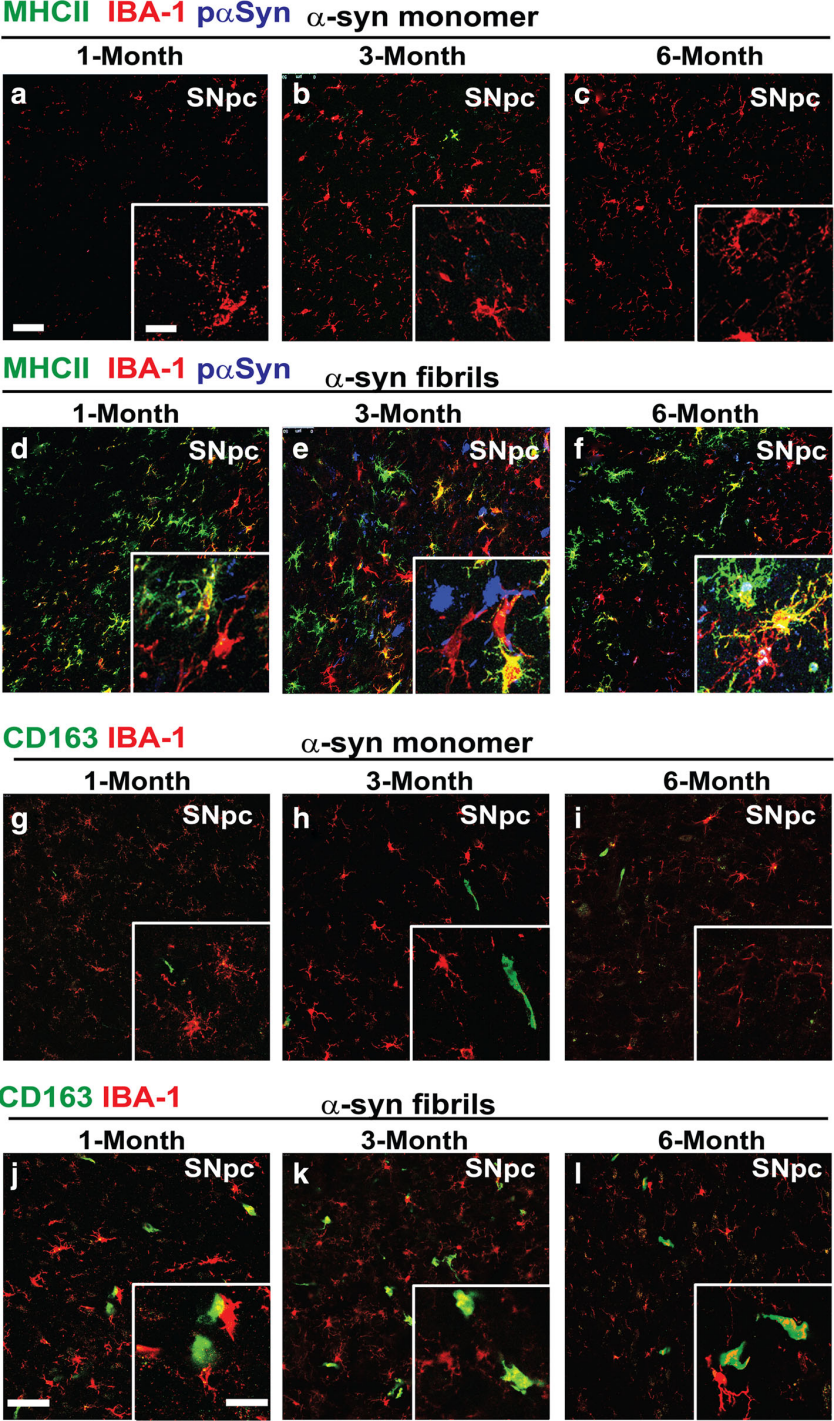
α-synuclein fibrils, but not monomer exposures, result in a sustained MHCII and CD163+ cell response in the SNpc. Representative confocal images of the rat SNpc (coronal sections). MHCII (green color), IBA-1 (red color) and pSer129-α-synuclein (blue color) signal was evaluated in monomer-injected rats 1 month (**a**), 3 months (**b**), and 6 months (**c**) post injection, and in fibril-injected rats 1 month (**d**), 3 months (**e**), and 6 months (**f**) post injection. CD163 stain (green color) with IBA-1 (red color) was evaluated in serial sections from the same rats (panels **g-l**). CD163+ cells shown are outside of the vasculature and therefore not perivascular macrophages. Scale bar is 30 μm, and white boxes are zoom insets with a scale bar of 10 μm. Images are representative of the SNpc in at least three rats analyzed per group

In vitro experiments with primary microglia suggest monomeric α-synuclein poorly induces pro-inflammatory responses compared to fibrillar α-synuclein. We further probed the monomer and fibril injected rats by evaluating the SNpc injection site one, three, and six months later. In contrast to fibril-injected rats, monomer exposures did not lead to a sustained MHCII expression in the SNpc (or saline-only) injections (Fig. 3a-f). CD163-positive cells in the monomer-injected SNpc could be detected only in the vasculature, indicative of the normal presence of perivascular macrophages (Fig. 3g-i). In contrast, CD163-positive cells were detected in the SNpc distant from any vasculature in fibril-injected rats at 1, 3 and 6 months post-injection (Fig. 3j-l). Efforts to further probe the surface markers of these MHCII expressing cells using antibodies to TMEM119 and CX3CR1 were unsuccessful due to a lack of antibody efficacy and specificity in rats compared to mice where the antibodies work well (Additional file 1: Figure S1). Overall, these results indicate that the inflammatory response observed at twenty-four hours with monomeric α-synuclein protein had resolved, whereas α-synuclein fibrils cause a more persistent MHCII expression that may involve peripheral immune cells.

### α-Synuclein fibrils promote microglial activation (but not microgliosis) with peripheral immune cell infiltration in the SNpc

**Fig. 4:**
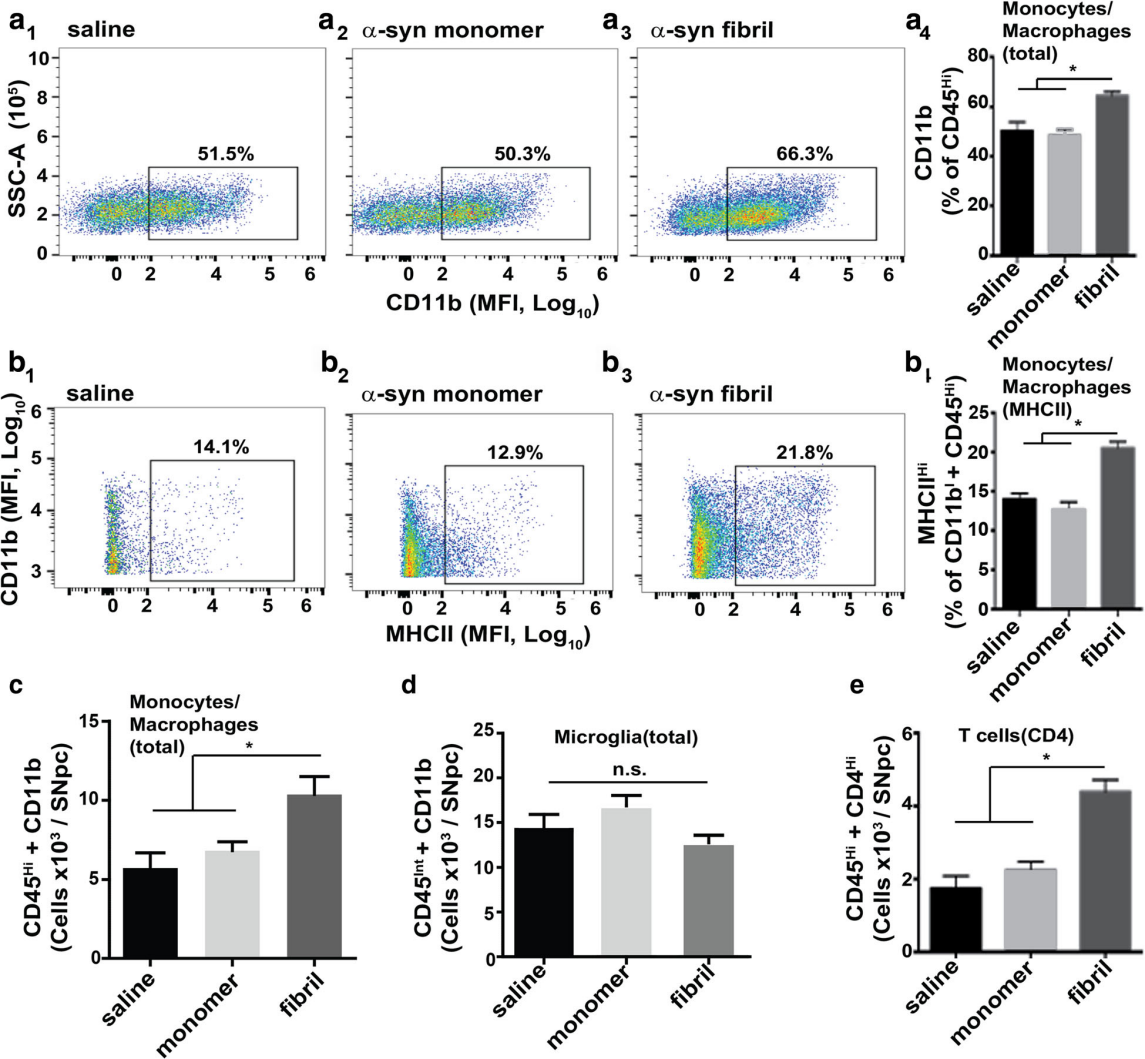
α-Synuclein fibrils promote microglial activation (but not proliferation) and peripheral immune cell infiltration in the SNpc. **a** Flow cytometry analysis of CD45^hi^, CD11b-expressing monocytes and macrophages in ventral midbrain isolates from 45 rats injected with either saline (A1), monomer (A2), or α-synuclein fibrils (A3), bi-laterally, eight-weeks post injection. (A4) Analysis of monocytes and macrophages based on CD45^hi^ expression in live cells (see Additional file 1: Figure S2 for full gating strategy). **b** Flow cytometry analysis of CD45^hi^, CD11b, MHCII positive cells (inflammatory monocytes and macrophages) in ventral midbrain isolates from saline (B1), monomer (B2), and α-synuclein fibril (B3) injected rats. (B4) Quantification of high MHCII-expressing cells in the monocyte and macrophage pool. **c** Quantification of monocytes/macrophages in ventral midbrain isolates, determined by CD45^Hi^ and CD11b expression. **d** Quantification of the number of CD45^int^, CD11b expressing microglia in ventral midbrain isolates. **e** Quantification of CD4-expressing T cells. Graphs show columns with mean values and error bars representing SEM. N=15 rats analyzed per group. *p<0.05, Tukey’s post hoc test. n.s. is not significant (one-way ANOVA). Scatterplots shown are representative of the groups

Our confocal analyses have identified two features that distinguish fibril-α-synuclein effects from monomer effects: recruitment of CD163-positive monocytes and macrophages and sustained MHCII responses in fibril, but not monomer, injected rats. To quantify these processes, we developed a protocol for mononuclear cell isolation from microdissected rat brain tissue and subsequent flow cytometry analysis from matched saline, monomer, and fibril injected rats, two-months post-injection. Cells were first gated for CD45 and CD11b expression to select myeloid lineages (gating strategy presented in Additional file 1: Figure S2). Measurements of CD11b expressing CD45^hi^ cells that mark infiltrating monocytes and macrophages [5] revealed significant increases with fibril exposures as compared to monomer or saline injections (Fig. 4). Monomer and saline controls were similar in all our measures, showing that the effects of the surgical procedure and monomeric α-synuclein protein (and co-contaminants inherent to the α-synuclein protein preparation) resolved and could not be distinguished two-months after injections (Fig. 4a).

To determine if the population of monocytes and macrophages from the periphery were in-part responsible for the enhanced MHCII expression in the SNpc we could observe by confocal analysis, we gated CD11b expressing and CD45^hi^ cells for MHCII expression using the same MHCII antibody clone used for confocal analysis. Fibril exposures approximately doubled the number of CD45^hi^ and CD11b-expressing cells, and those that expressed the highest MHCII levels (Fig. 4b, c). The total number of microglia did not change compared to saline or monomer exposures, suggesting a lack of microglia proliferation or changes in turn-over (Fig. 4d). The overall increase in MHCII positive cells was therefore due in-part to infiltrating monocytes and macrophages.

Finally, we analyzed the SNpc cell isolate for CD4 positive T-cells of the adaptive immune system and again detected a robust increase in peripheral immune cells in fibril-injected rats as compared to monomer or saline-only injections (Fig. 4e). While T-cells lack MHCII expression, they corroborate the exacerbated and sustained pro-inflammatory response defined by MHCII expression and peripheral cell recruitment caused by α-synuclein fibril exposures.

### α-Synuclein fibril exposures in the SNpc promote pSer129-α-synuclein inclusions, progressive loss of tyrosine-hydroxylase (TH) expression, and striatal degeneration

**Fig. 5:**
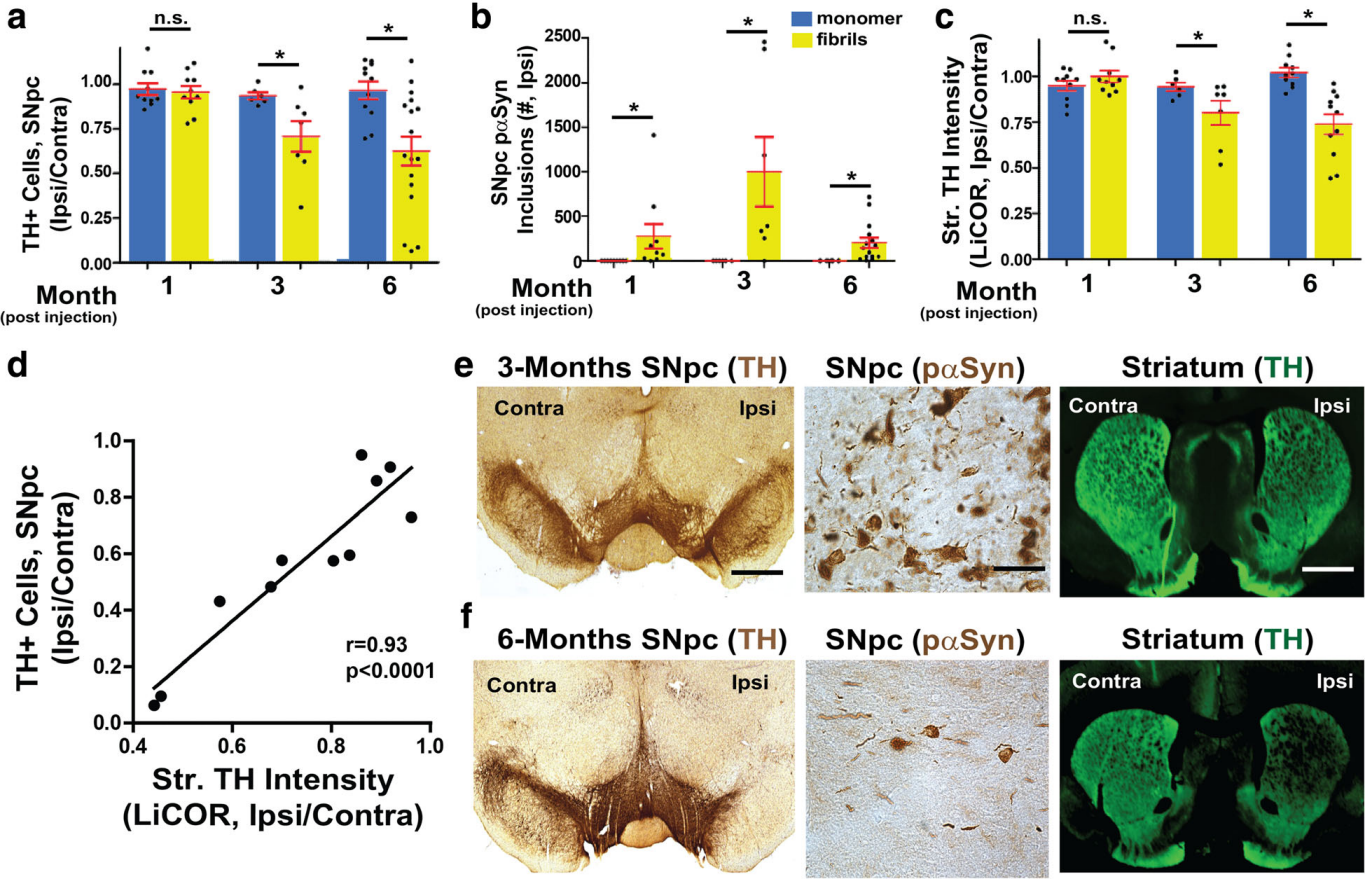
α-Synuclein fibril exposures in the SNpc promote pSer129-α-synuclein inclusions, progressive dopaminergic cell loss and striatal degeneration. Quantification of TH-expression and inclusions over time in 60 rats unilaterally injected with either monomer or α-synuclein fibrils, analyzed one to six-months post-injection. **a** Unbiased stereological estimates of TH-expressing neurons in the SNpc (normalized to un-injected contralateral SNpc) at one month, three months, and six months post injection. Data from monomer-injected rats are plotted in blue and fibrils in yellow. **b** Unbiased stereological estimates of pSer-129-α-synuclein inclusions in the ipsilateral SNpc. **c** Striatal TH-fiber density measured by TH immunofluorescence using LiCOR, with ratios presented from signal in ipsilateral-injected dorsal striatum divided by contralateral (uninjected) dorsal striatum. **d** Comparison plot showing the correlation of neuronal cell loss in the SNpc with striatal fiber density. Pearson’s r = 0.93, p<0.0001. **e** Representative images for TH and p-Ser129-α-synuclein immunohistochemistry at 3 months and **f** six-months post injection with α-synuclein fibrils. Scale bars are 0.5 mm and 50 μm (high-mag panels). Column graphs show group mean values and error bars show SEM. Data points represent mean values from individual rats. *p<0.05, Tukey’s post-hoc test and one-way ANOVA

Confocal analyses revealed sparse or no phosphorylated α-synuclein inclusions one-month post fibril injection, whereas robust inclusions in TH-expressing cells in the SNpc could be detected three and six-months post-fibril injection (Fig. 3a-f). To quantify inclusion formation and loss of TH-expression in the context of persistent neuroinflammation, serial sections of the rat SNpc processed for MHCII and CD163 staining were also analyzed via unbiased stereology for TH-expressing neurons at one, three, and six months’ post-injection. We found a significant loss of TH-positive cells three and six-months post injection (Fig. 5a). No significant change in the numbers of TH-positive neurons were detected in the monomer injected SNpc between 1, 3, and 6 months (average 8779±323 neurons, n=25). In contrast, the number of TH-positive neurons in the fibril-injected SNpc reduced from 1 to 3 to 6 months (average 6697±341, 6213±1303, and 4426±688, respectively, n=34). The number of TH-positive neurons in the un-injected SNpc was similar between all groups (cumulative average 8129±257, n=59).

The number of inclusions in the SNpc peaked at three-months with thousands of inclusions detected in some rats, whereas hundreds of inclusions were detected across the SNpc at one and six-months post fibril injection (Fig. 5b). pSer129-α-synuclein inclusions were never detected in any of the monomer-injected rats. To determine the viability of dopaminergic nigro/striatal projections at these time-points, striatal TH fiber density in the dorsal striatum was measured via LiCOR tissue imaging and decreased densities could be detected three and six months’ post α-syn fibril injections (Fig. 5c). Decreased TH fiber density correlated well (r=0.93) with decreases in the numbers of TH-expressing neurons in the SNpc (Fig. 5d). The timing of neurodegeneration and inclusion distribution with SNpc fibril injections is therefore different than previous reports of fibril injections in the dorsal striatum in rats [29].

### α-Synuclein fibril exposure in the SNpc induces progressive inflammation and inclusion formation in the dorsal striatum

**Fig. 6:**
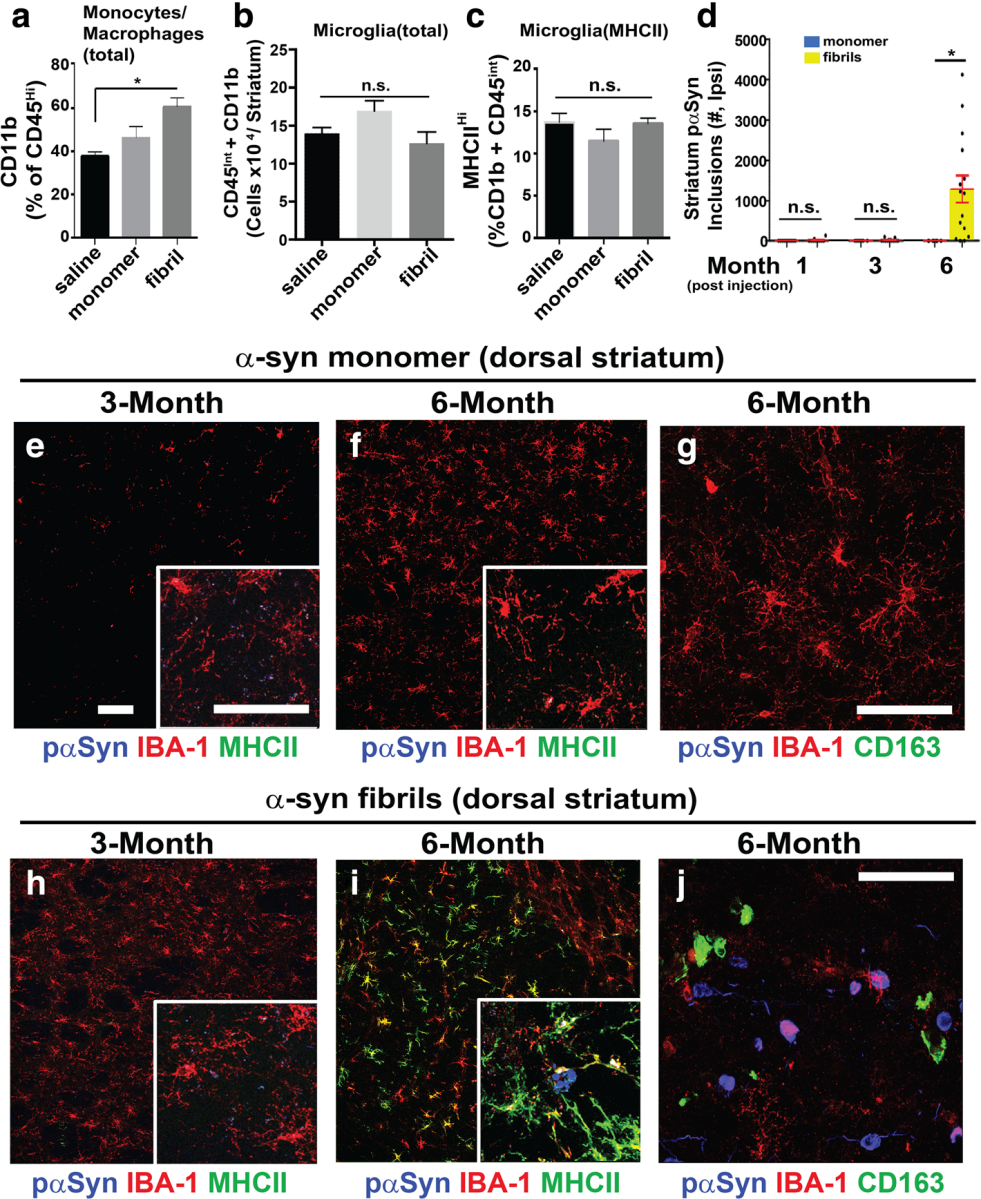
α-Synuclein fibril exposure in the SNpc induces progressive inflammation and inclusion formation in the dorsal striatum. **a** Flow cytometry analysis of live CD45^hi^, CD11b-expressing monocytes and macrophages in dorsal striatum tissue isolates from 45 rats bi-laterally injected with either saline, monomer, or α-synuclein fibrils and analyzed eight-weeks post injection. **b** Quantification of microglia, eight-weeks post-injection, and those that are (**c**) expressing MHCII in the dorsal striatum. Scatter plots are presented in Additional file 1: Figure S3. **d** Unbiased stereological estimates of p-Ser129-α-synuclein inclusions in the ipsilateral dorsal striatum one, three, and six months post-injection monomer (blue bars) or fibrils (yellow bars) injections. Data from 60 rats is shown. **e-g** From the rats analyzed in panel **d**, representative immunofluorescence depicting inflammation in the ipsilateral dorsal striatum caused by monomer injections or (**h-j**) fibril injections. Confocal images are shown with MHCII or CD163 as green color, IBA-1 as red color, and pSer129-α-synuclein as blue color. Scale bars are 40 μm, with white boxes showing zoom insets. Column graphs show group mean values and error bars show SEM. Data points represent mean values from individual rats. *p<0.05, Tukey’s post-hoc test and one-way ANOVA. n.s. is not significant

A main feature of fibril injections in the rat SNpc is that inclusions eventually spread to spiny projection neurons in the dorsal striatum and this correlates with dopaminergic neurodegeneration [1]. To determine whether α-synuclein fibrils introduced in the SNpc induce inflammation in the dorsal striatum, we analyzed tissue dissected from the striatum via flow cytometry. Two-months post fibril injection, we detected an increase in the number of monocytes and macrophages in the striatum, without an increase in MHCII expression, in fibril-injected rats (Fig. 6a, Additional file 1: Figure S3). The number of microglia in the striatum 2-months post-fibril injection were unchanged in all conditions (Fig. 6b, c).

Stereological counts of inclusions through the striatum revealed that inclusion spread from the SNpc to the striatum was a slow process where inclusion loads could only be detected at 6-months post-injection (Fig. 6d). Concomitant with the formation of new α-synuclein inclusions in the dorsal striatum, MHCII expression and CD163-cell recruitment in the striatum became robust six-months post-fibril injection in the SNpc in comparison to monomer-injected rats (Fig. 6e-j). These results demonstrate a marked pro-inflammatory response that spreads from the initial site of α-synuclein fibril injection in the SNpc to the striatum, with full MHCII expression and peripheral cell recruitment that may depend on the availability of local α-synuclein inclusions and fibrils (Fig. 6i, j).

## Discussion

**Fig. 7:**
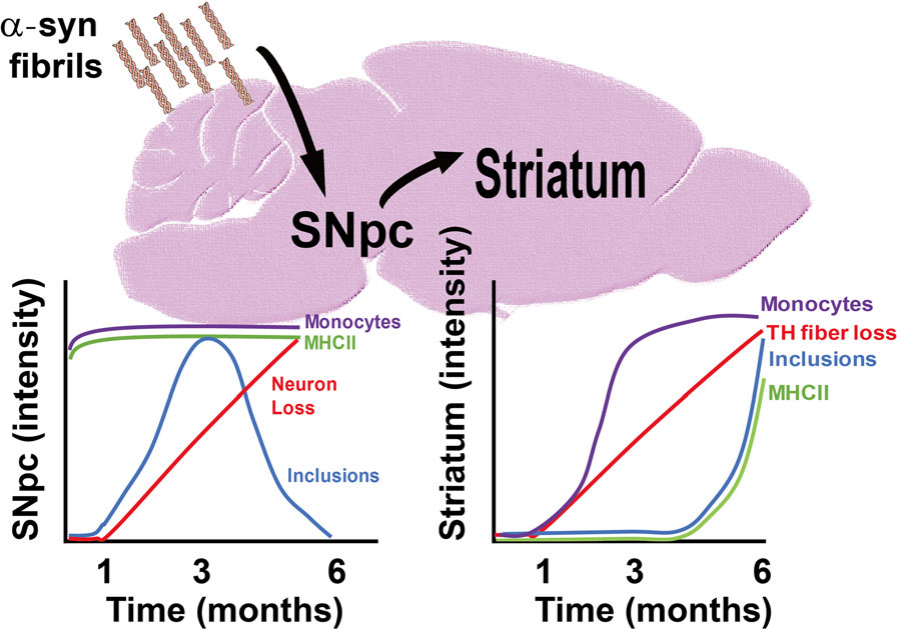
Interpretative model of changes over time in the rat SNpc fibril-injection model between monocytes (purple), MHCII expression (green), TH cell loss (red), and α-synuclein inclusions (blue)

Our observations center on three novel results in a newly developed rat model of α-synuclein-mediated neurodegeneration: First, MHCII induction and peripheral monocyte and macrophage recruitment occurs shortly after initial fibril exposure in the SNpc. The recruitment of peripheral innate immune cells does not occur with equivalent amounts of monomeric α-synuclein, possibly owing to α-synuclein fibrils possessing much higher (w/v) pro-inflammatory agonist potential as we could record in cultured microglia. Second, MHCII induction is persistent over time in the SNpc despite the eventual loss of TH-expression and local neuronal inclusions. Third, MHCII-expressing cells eventually spread to the dorsal striatum commensurate with the formation of inclusions in striatal neurons. The loss of TH-fibers in the striatum and fibril exposures was apparently not enough to cause MHCII that may need α-synuclein fibrils, or neurons that harbor them, nearby for induction. Together, these results demonstrate that MHCII activation occurs prior to neurodegeneration and can potentially be explained by potent agonist activity of fibrillar α-synuclein that involves activation of both resident and peripheral leukocytes and lymphocytes (Fig. 7).

MHCII activation in the PD brain has been known for decades[26] but very difficult to understand. Based on morphologies of the MHCII cell population, activation was considered a generalized property of microgliosis (e.g., the expansion of resident pools of microglia) common to most neurodegenerative diseases. Whether MHCII induction played a detrimental, protective, or benign role with respect to disease susceptibility and progression cannot be easily understood from post-mortem tissue [7]. More recent genetic studies have resolved part of these issues by assigning a certain causative role for both MHCII proteins (HLA-DR) [15] and α-synuclein (SNCA) [25, 32, 35, 36] in mediating major parts of the heritable aspects of PD. In contrast to α-synuclein, MHCII expression is largely restricted to cells of the innate immune system, occurring in both resident cells in tissue like microglia [16] as well as cells in peripheral cells in circulation like monocytes [18]. The combination of genetic and pathological studies therefore suggests an important interaction between α-synuclein and MHCII expression that might be exploited for therapeutic gain.

In this study, we analyzed MHCII expression on microglia and infiltrating monocytes and macrophages using immunofluorescence approaches and flow cytometry experiments. As compared to mouse models, tools available to definitively delineate infiltrating myeloid cells like monocytes and macrophages from resident activated cells like microglia are somewhat lacking in rat models. Microglial-specific markers that are routinely used in mouse tissues such as TMEM119, CX3CR1, and P2ry12 [2, 4, 43] were unfortunately not effective in labeling rat immune cells likely due to epitope differences between mice and rats (Additional file 1: Figure S1). For immunoflouresence analysis, we relied largely on CD163 with weaker IBA-1 and amoeboid morphology to identify macrophages, consistent with past studies [2, 13, 40]. In human post-mortem tissue, there is some evidence the CD163 scavenger receptor may be expressed in microglia [30], although in the rats included in this study we did not observe these morphological characteristics reminiscent of microglia in CD163-positive cells. It is important to note that CD163 can also label perivascular macrophages. In our analyses, confocal analysis was isolated to areas that lacked perivascular spaces.

To corroborate our approaches, flow-cytometry was used to separate monocytes and macrophages from resident microglia. Gating on CD45 expression, we could confirm our immunofluorescence observations in showing that α-synuclein fibrils recruit monocytes and macrophages from the periphery. CD45 expression, whilst routinely used to differentiate microglia from monocytes and macrophages, can change in unexpected ways with active disease states. It is possible that a portion, or all, of our CD45-centric flow-cytometry analysis has identified a subset of cells derived from microglia and not peripheral monocytes as conventional wisdom would suppose. Nevertheless, our overall conclusion that α-synuclein fibrils recruit peripheral immune cells is conclusive since we also detect robust CD4+ T cells correlated with MHCII-induction in both the SNpc and striatum. While the results from this study clearly indicate a peripheral immune response, future studies are warranted using transgenic and knockout models to determine the role and timing of infiltrating monocytes, macrophages, and T cells in response to fibril exposures and spread.

While MHCII is unambiguously upregulated in PD-affected brains [26], whether peripheral cells contribute to the MHCII pool in the human brain has been a matter of intense debate. As a canonical target of the type II interferon signaling pathway, MHCII expression is linked to pro-inflammatory responses in innate immune cells, different from healing responses that can also be associated with these cells [28]. Recent studies demonstrate that both innate and adaptive immune cells from the periphery constantly survey the brain as in important part of brain function [23]. Here, we can show that lasting peripheral immune cell recruitment to the brain and MHCII induction does not occur via saline-only or monomer α-synuclein injections, but occurs with α-synuclein fibril exposures. Since fibrils compose the inclusions in PD and related synucleinopathies, we hypothesize that a major part of the toxicity related to inclusion formation is the recruitment of peripheral immune cells that express pro-inflammatory markers like MHCII.

In isolated microglia following α-synuclein fibril treatment, we found significant expression of chemokines responsible for peripheral immune cell recruitment including CXCL10, CCL2, MIP-1α, MIP-1β, and MIP-2. These results indicate a classical innate immune response initiated by fibril exposures that result in recruitment of monocytes, macrophages, and T cells. While these data indicate microglia as part of the source for the chemokines that recruit immune cells, future studies are warranted to determine all the different contributions of this process and whether strategies that block peripheral cell recruitment are protective in this model.

An important limitation to our study is that we cannot rule out synergistic effects of blood-brain-barrier disruption due to the surgery combined with the enhanced agonist potential of α-synuclein fibrils compared to monomeric protein. Another limitation is that we were unable to separate immunological phenotypes due to the fibrils we injected versus those that subsequently formed in neurons. Future studies that involve fibril-exposures in the periphery that eventually spread to the brain over many months or years, together with α-synuclein knockout rodents that cannot intrinsically form new inclusions, would be informative in resolving both limitations. We suspect that monocytes and macrophages would gain access to neurons harboring α-synuclein fibrils whether or not the blood-brain-barrier is mechanically disrupted, based on the analysis of striatum tissue in our study. Further, based on the time course of analysis of both the SNpc and the striatum in our study, we suspect that sustained MHCII expression is contingent only on the induction of inclusions in neurons, not on the initial fibrils we injected or subsequent neurodegeneration. Future studies are warranted to test these hypotheses.

In isolated microglia, we measured uptake of antigen and presentation. α-Synuclein fibrils but not monomeric protein induces MHCII *in vitro* and *in vivo*, setting in motion a cascade for possible MHCII presentation of antigen to T helper cells that activate the adaptive immune response. These T-cells were readily detectable by flow cytometry in the brain, but unfortunately, we did not find antibodies for rat T-cells that were compatible with confocal analysis. Recent data demonstrate that neurons may be concurrently expressing α-synuclein peptides via MHCI to T cells [38]. The persistence of MHCII expression that can be observed in both our rat model and in post-mortem PD tissue is still mysterious, although one explanation is that MHCII upregulation in microglia may draw in peripheral cells and both MHCI and MHCII over-rides counter anti-inflammatory responses that would otherwise allow for resolution.

While MHCII typifies responses that usually protect hosts from pathogens, the inappropriate processing of α-synuclein fibrils through this pathway may induce damaging pro-inflammatory cytokine responses like IFN and reactive oxygen species (ROS) production. The degradation and processing of fibrils that occurs in innate immune cells necessarily reduces the number of fibrils in the brain, which may be beneficial with respect to fibril spread, but at a large cost of concurrent cytokine and ROS production that may damage neurons.

In the model studied here, MHCII expression derives from both monocytes and macrophages and microglia, and besides CD163 expression that identifies these cells, peripheral immune cells may lack signaling pathways inherent in microglia that allow interactions with neurons to quell pro-inflammatory responses. Fractalkine signaling is one such mechanism inherent to microglia but not monocytes and macrophages [27]. Based on the activation profiles of the monocytes and macrophage and T-cells we could detect in our rat model, we further hypothesize that these peripheral immune cells are unlikely to be associated with a healing response and are unlikely to be beneficial. Based on these observations, it seems reasonable to propose studies that determine whether blocking the recruitment of monocytes and T-cells to the brain slows neurodegeneration associated with α-synuclein fibril exposures.

## Conclusions

α-Synuclein fibrils stimulate chemokines and MHCII induction in microglia, and peripheral monocyte and macrophage recruitment occurs shortly after fibril exposures in the brain. MHCII induction persists over time, and eventually spreads to other parts of the brain concomitant with inclusion spread. In this model, peripheral immune cells contribute to MHCII induction that occurs after abnormal α-synuclein is present but before neurodegeneration.

## Additional file

Additional file 1: Supplemental figures. (PDF 31368 kb)

## Abbreviations

CD11b: Integrin, alpha-M
CD163: Hemoglobin scavenger receptor
CD4: T-cell antigen T4
CD45: Leukocyte-common antigen
CX3CR1: Fractalkine receptor
G-CSF: granulocyte-colony stimulating factor
HLA-DR: Human leukocyte antigen – antigen D related
IBA-1: Allograft inflammatory factor 1
IgG1: Immunoglobulin G1
IL-17: interleukin-17
IL-1α: interleukin-1 a
iNOS: inducible nitric oxide synthase
MHCII: major histocompatibility complex type II
MPTP: 1-methyl-4-phenyl-1,2,3,6-tetrahydropyridine
NeuN: Neuronal nuclei-binding
P2ry12: Purinoreeptor P2Y12
PD: Parkinson disease
SNpc: Substantia Nigra pars compacta
TH: Tyrosine hydroxylase
TMEM119: Transmembrane protein 119
TNF: tumor necrosis factor

## References

[1] Abdelmotilib H, Maltbie T, Delic V, Liu Z, Hu X, Fraser KB, Moehle MS, Stoyka L, Anabtawi N, Krendelchtchikova V (2017). alpha-Synuclein fibril-induced inclusion spread in rats and mice correlates with dopaminergic Neurodegeneration. Neurobiol Dis.

[2] Bennett ML, Bennett FC, Liddelow SA, Ajami B, Zamanian JL, Fernhoff NB, Mulinyawe SB, Bohlen CJ, Adil A, Tucker A (2016). New tools for studying microglia in the mouse and human CNS. Proc Natl Acad Sci U S A.

[3] Bousset L, Brundin P, Bockmann A, Meier B, Melki R (2016). An Efficient Procedure for Removal and Inactivation of Alpha-Synuclein Assemblies from Laboratory Materials. J Parkinsons Dis.

[4] Butovsky O, Jedrychowski MP, Moore CS, Cialic R, Lanser AJ, Gabriely G, Koeglsperger T, Dake B, Wu PM, Doykan CE (2014). Identification of a unique TGF-beta-dependent molecular and functional signature in microglia. Nature neuroscience.

[5] Carson MJ, Reilly CR, Sutcliffe JG, Lo D (1998). Mature microglia resemble immature antigen-presenting cells. Glia.

[6] Cebrian C, Zucca FA, Mauri P, Steinbeck JA, Studer L, Scherzer CR, Kanter E, Budhu S, Mandelbaum J, Vonsattel JP (2014). MHC-I expression renders catecholaminergic neurons susceptible to T-cell-mediated degeneration. Nat Commun.

[7] Croisier E, Moran LB, Dexter DT, Pearce RK, Graeber MB (2005). Microglial inflammation in the parkinsonian substantia nigra: relationship to alpha-synuclein deposition. J Neuroinflammation.

[8] Czlonkowska A, Kurkowska-Jastrzebska I, Czlonkowski A (2000). Inflammatory changes in the substantia nigra and striatum following MPTP intoxication. Ann Neurol.

[9] Delgado-Alvarado M, Gago B, Gorostidi A, Jimenez-Urbieta H, Dacosta-Aguayo R, Navalpotro-Gomez I, Ruiz-Martinez J, Bergareche A, Marti-Masso JF, Martinez-Lage P (2017). Tau/alpha-synuclein ratio and inflammatory proteins in Parkinson's disease: An exploratory study. Mov Disord.

[10] Dufek M, Rektorova I, Thon V, Lokaj J, Rektor I (2015). Interleukin-6 May Contribute to Mortality in Parkinson's Disease Patients: A 4-Year Prospective Study. Parkinsons Dis.

[11] Fellner L, Irschick R, Schanda K, Reindl M, Klimaschewski L, Poewe W, Wenning GK, Stefanova N (2013). Toll-like receptor 4 is required for alpha-synuclein dependent activation of microglia and astroglia. Glia.

[12] Fujiwara H, Hasegawa M, Dohmae N, Kawashima A, Masliah E, Goldberg MS, Shen J, Takio K, Iwatsubo T (2002). alpha-Synuclein is phosphorylated in synucleinopathy lesions. Nat Cell Biol.

[13] Goldmann T, Wieghofer P, Jordao MJ, Prutek F, Hagemeyer N, Frenzel K, Amann L, Staszewski O, Kierdorf K, Krueger Met al (2016) Origin, fate and dynamics of macrophages at central nervous system interfaces. Nat Immunol 17:797-805 doi:10.1038/ni.3423 10.1038/ni.3423 PMC4968048 27135602

[14] Gustot A, Gallea JI, Sarroukh R, Celej MS, Ruysschaert JM, Raussens V (2015). Amyloid fibrils are the molecular trigger of inflammation in Parkinson's disease. Biochem J.

[15] Hamza TH, Zabetian CP, Tenesa A, Laederach A, Montimurro J, Yearout D, Kay DM, Doheny KF, Paschall J, Pugh E (2010). Common genetic variation in the HLA region is associated with late-onset sporadic Parkinson's disease. Nat Genet.

[16] Harms AS, Cao S, Rowse AL, Thome AD, Li X, Mangieri LR, Cron RQ, Shacka JJ, Raman C, Standaert DG (2013). MHCII is required for alpha-synuclein-induced activation of microglia, CD4 T cell proliferation, and dopaminergic neurodegeneration. J Neuroscience : Official J Soc Neuroscience.

[17] Imamura K, Hishikawa N, Sawada M, Nagatsu T, Yoshida M, Hashizume Y (2003). Distribution of major histocompatibility complex class II-positive microglia and cytokine profile of Parkinson's disease brains. Acta Neuropathol.

[18] Jakubzick C, Gautier EL, Gibbings SL, Sojka DK, Schlitzer A, Johnson TE, Ivanov S, Duan Q, Bala S, Condon T (2013). Minimal differentiation of classical monocytes as they survey steady-state tissues and transport antigen to lymph nodes. Immunity.

[19] Jimenez-Ferrer I, Jewett M, Tontanahal A, Romero-Ramos M, Swanberg M (2017). Allelic difference in Mhc2ta confers altered microglial activation and susceptibility to alpha-synuclein-induced dopaminergic neurodegeneration. Neurobiol Dis.

[20] Kim C, Ho DH, Suk JE, You S, Michael S, Kang J, Joong Lee S, Masliah E, Hwang D, Lee HJ (2013). Neuron-released oligomeric alpha-synuclein is an endogenous agonist of TLR2 for paracrine activation of microglia. Nat Commun.

[21] Kim C, Lee HJ, Masliah E, Lee SJ (2016). Non-cell-autonomous Neurotoxicity of alpha-synuclein Through Microglial Toll-like Receptor 2. Exp Neurobiol.

[22] Langston JW, Forno LS, Tetrud J, Reeves AG, Kaplan JA, Karluk D (1999). Evidence of active nerve cell degeneration in the substantia nigra of humans years after 1-methyl-4-phenyl-1,2,3,6-tetrahydropyridine exposure. Ann Neurol.

[23] Louveau A, Smirnov I, Keyes TJ, Eccles JD, Rouhani SJ, Peske JD, Derecki NC, Castle D, Mandell JW, Lee KS (2015). Structural and functional features of central nervous system lymphatic vessels. Nature.

[24] Luk KC, Kehm V, Carroll J, Zhang B, O'Brien P, Trojanowski JQ, Lee VM (2012). Pathological alpha-synuclein transmission initiates Parkinson-like neurodegeneration in nontransgenic mice. Science.

[25] Maraganore DM, de Andrade M, Elbaz A, Farrer MJ, Ioannidis JP, Kruger R, Rocca WA, Schneider NK, Lesnick TG, Lincoln SJet al (2006) Collaborative analysis of alpha-synuclein gene promoter variability and Parkinson disease. JAMA : the journal of the American Medical Association 296: 661-670 doi:10.1001/jama.296.6.661 10.1001/jama.296.6.661 16896109

[26] McGeer PL, Itagaki S, Boyes BE, McGeer EG (1988). Reactive microglia are positive for HLA-DR in the substantia nigra of Parkinson's and Alzheimer's disease brains. Neurology.

[27] Mizutani M, Pino PA, Saederup N, Charo IF, Ransohoff RM, Cardona AE (2012). The fractalkine receptor but not CCR2 is present on microglia from embryonic development throughout adulthood. Journal of immunology.

[28] Moehle MS, West AB (2015). M1 and M2 immune activation in Parkinson's Disease: Foe and ally?. Neuroscience.

[29] Paumier KL, Luk KC, Manfredsson FP, Kanaan NM, Lipton JW, Collier TJ, Steece-Collier K, Kemp CJ, Celano S, Schulz Eet al (2015) Intrastriatal injection of pre-formed mouse alpha-synuclein fibrils into rats triggers alpha-synuclein pathology and bilateral nigrostriatal degeneration. Neurobiol Dis 82: 185-199 doi:10.1016/j.nbd.2015.06.003 10.1016/j.nbd.2015.06.003 PMC4640952 26093169

[30] Pey P, Pearce RK, Kalaitzakis ME, Griffin WS, Gentleman SM (2014). Phenotypic profile of alternative activation marker CD163 is different in Alzheimer's and Parkinson's disease. Acta Neuropathol Commun.

[31] Polfliet MM, Fabriek BO, Daniels WP, Dijkstra CD, van den Berg TK (2006). The rat macrophage scavenger receptor CD163: expression, regulation and role in inflammatory mediator production. Immunobiology.

[32] Polymeropoulos MH, Lavedan C, Leroy E, Ide SE, Dehejia A, Dutra A, Pike B, Root H, Rubenstein J, Boyer R (1997). Mutation in the alpha-synuclein gene identified in families with Parkinson's disease. Science.

[33] Qin H, Buckley JA, Li X, Liu Y, Fox TH, 3rd, Meares GP, Yu H, Yan Z, Harms AS, Li Yet al (2016) Inhibition of the JAK/STAT Pathway Protects Against alpha-Synuclein-Induced Neuroinflammation and Dopaminergic Neurodegeneration. J Neurosci 36: 5144-5159 doi:10.1523/JNEUROSCI.4658-15.2016 10.1523/JNEUROSCI.4658-15.2016 PMC6123006 27147665

[34] Sanchez-Guajardo V, Febbraro F, Kirik D, Romero-Ramos M (2010). Microglia acquire distinct activation profiles depending on the degree of alpha-synuclein neuropathology in a rAAV based model of Parkinson's disease. PLoS One.

[35] Singleton AB, Farrer MJ, Bonifati V (2013). The genetics of Parkinson's disease: progress and therapeutic implications. Mov Disord.

[36] Soldner F, Stelzer Y, Shivalila CS, Abraham BJ, Latourelle JC, Barrasa MI, Goldmann J, Myers RH, Young RA, Jaenisch R (2016). Parkinson-associated risk variant in distal enhancer of alpha-synuclein modulates target gene expression. Nature.

[37] Stefanova N, Fellner L, Reindl M, Masliah E, Poewe W, Wenning GK (2011). Toll-like receptor 4 promotes alpha-synuclein clearance and survival of nigral dopaminergic neurons. Am J Pathol.

[38] Sulzer D, Alcalay RN, Garretti F, Cote L, Kanter E, Agin-Liebes J, Liong C, McMurtrey C, Hildebrand WH, Mao X (2017). T cells from patients with Parkinson's disease recognize alpha-synuclein peptides. Nature.

[39] Thomzig A, Wagenfuhr K, Daus ML, Joncic M, Schulz-Schaeffer WJ, Thanheiser M, Mielke M, Beekes M (2014). Decontamination of medical devices from pathological amyloid-beta-, tau- and alpha-synuclein aggregates. Acta Neuropathol Commun.

[40] Utans U, Arceci RJ, Yamashita Y, Russell ME (1995). Cloning and characterization of allograft inflammatory factor-1: a novel macrophage factor identified in rat cardiac allografts with chronic rejection. J Clin Invest.

[41] Volpicelli-Daley LA, Luk KC, Lee VM (2014). Addition of exogenous alpha-synuclein preformed fibrils to primary neuronal cultures to seed recruitment of endogenous alpha-synuclein to Lewy body and Lewy neurite-like aggregates. Nat Protoc.

[42] Volpicelli-Daley LA, Luk KC, Patel TP, Tanik SA, Riddle DM, Stieber A, Meaney DF, Trojanowski JQ, Lee VM (2011). Exogenous alpha-synuclein fibrils induce Lewy body pathology leading to synaptic dysfunction and neuron death. Neuron.

[43] Yamasaki R, Lu H, Butovsky O, Ohno N, Rietsch AM, Cialic R, Wu PM, Doykan CE, Lin J, Cotleur ACet al (2014) Differential roles of microglia and monocytes in the inflamed central nervous system. J Exp Med 211: 1533-1549 doi:10.1084/jem.20132477 10.1084/jem.20132477 PMC4113947 25002752

