# Supplementary material for: α-Synuclein fibrils recruit peripheral immune cells in the rat brain prior to neurodegeneration
Source: Acta Neuropathol Commun. 2017 Nov 21;5:85. doi: 10.1186/s40478-017-0494-9 (PMC5698965; doi:10.1186/s40478-017-0494-9)
Supplement: Additional file 1: — Supplemental figures. (PDF 31368 kb) [file 40478_2017_494_MOESM1_ESM.pdf]

# α-Synuclein Fibrils Recruit Peripheral Immune cells in the Rat Brain Prior to Neurodegeneration

Ashley S. Harms*, Vedad Delic, Aaron D. Thome, Nicole Bryant, Zhiyong Liu, Sidhanth Chandra, Asta Jurkuvenaite, Andrew B. West

From the Center for Neurodegeneration and Experimental Therapeutics, Department of Neurology, University of Alabama at Birmingham, Birmingham, AL, 35294

*to whom correspondence should be addressed:
Email: anharms@uab.edu
Phone: 205-934-6142
Address: 1719 6th ave south, Birmingham, AL 35233

## Supplemental Figures

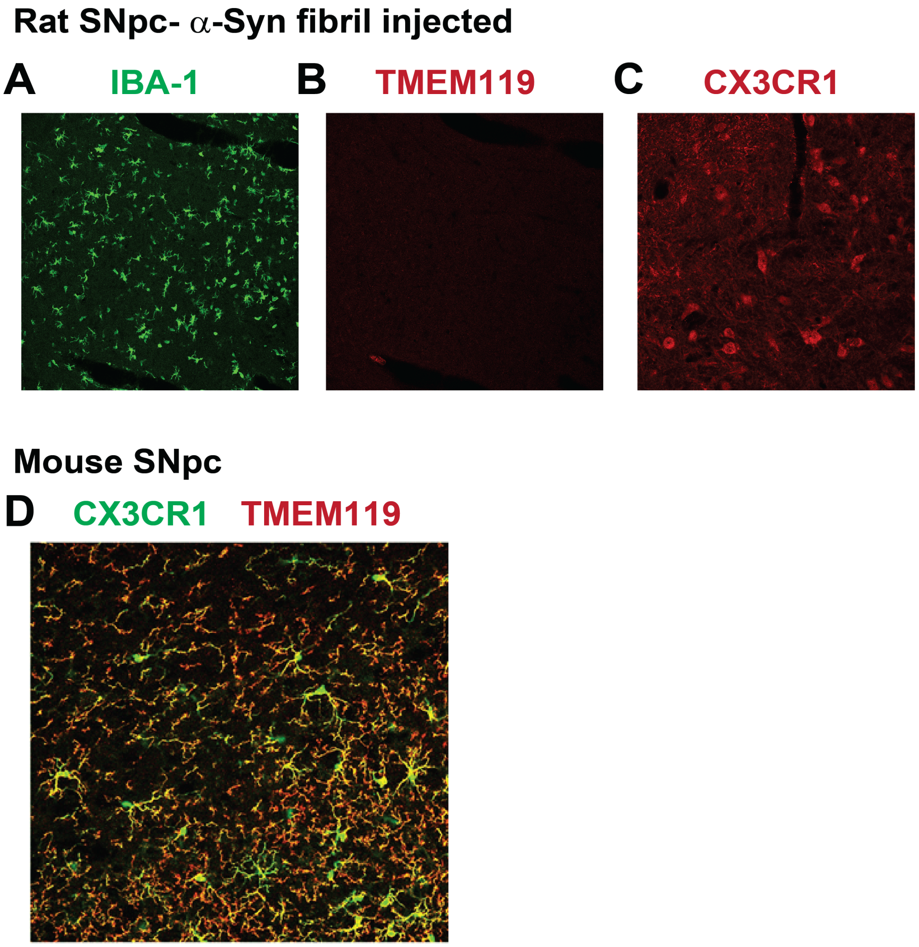


**Supplemental figure 1**. Representative confocal images of the rat SNpc (coronal sections), evaluated in fibril-injected rats 6-months post injection. (A) IBA-1 is shown in green, highlighting cells with morphology of microglia. (B) Antibodies to TMEM119 (monoclonal clone 28-3) failed to produce appreciable signal. (C) Antibodies to CX3CR1 (polyclonal Abcam, N-terminal epitope AA 2-21) cross-reacted with cells with the morphology and density of neurons in the SNpc, and did not show evidence of detecting microglia or myeloid cells. (D) In contrast, these antibodies demonstrated efficacy in labeling mouse microglia in the SNpc, from untreated WT- C57BL6J coronal sections.

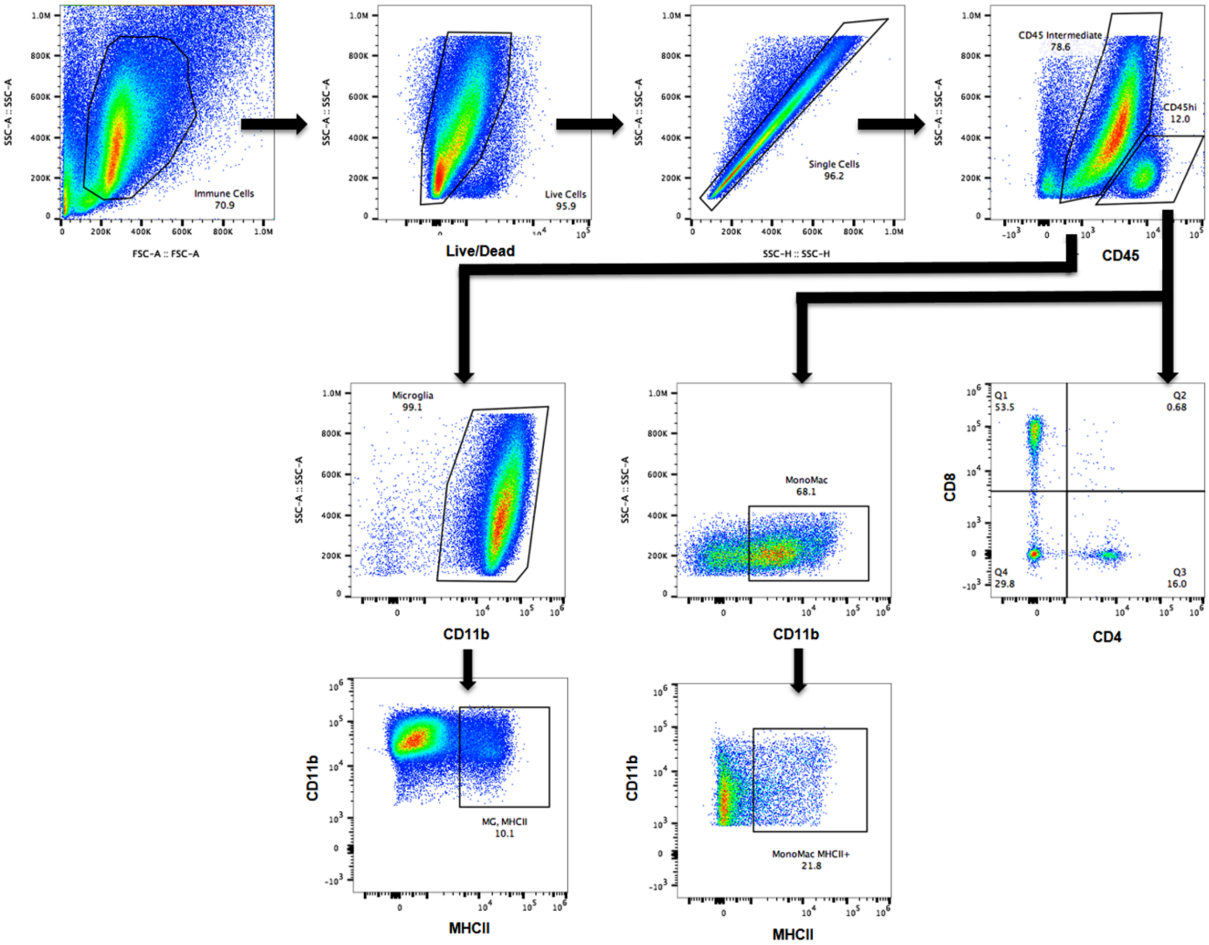


**Supplemental figure 2.** Overview of flow cytometry gating strategy.

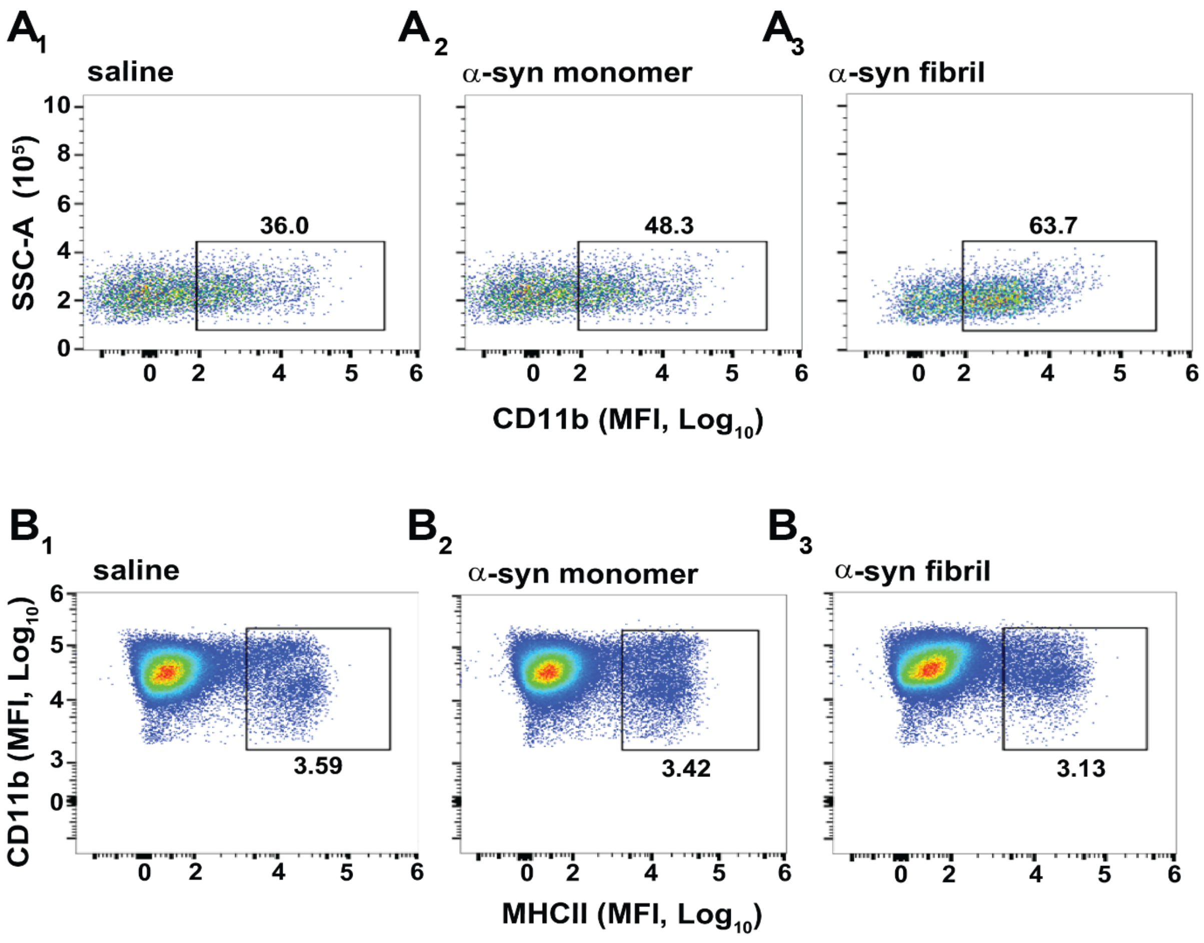


**Supplemental figure 3**. (A) Flow cytometry analysis of live CD45$^{hi}$, CD11b-expressing monocytes and macrophages in dorsal striatum tissue isolates from 45 rats bi-laterally injected with either saline, monomer, or α-synuclein fibrils and analyzed eight-weeks post injection. (B) MHCII expression in this cell population denotes pro-inflammatory polarizations.
